# Palestinian students’ attitudes toward honor killing crimes: a quantitative, cross-sectional study

**DOI:** 10.1186/s40359-025-03159-0

**Published:** 2025-08-04

**Authors:** Oqab Jabali, Bilal Hamamra, Abed Alkarim Ayyoub

**Affiliations:** 1 Language Center, Faculty of Humanities and Educational Sciences, An-Najah National University, Nablus, Palestine; 2 Department of English, Faculty of Humanities and Educational Sciences, An-Najah National University, Nablus, Palestine; 3 Department of Teachers’ Training, Faculty of Humanities and Educational Sciences An-Najah National University, Nablus, Palestine

**Keywords:** Honor killing, Students’ attitudes, Palestine, Legal system, Religion, Traditions

## Abstract

**Background:**

Honor killings are a deeply ingrained practice within Palestinian patriarchal culture, where violations of perceived family honor—particularly by women—can lead to extreme consequences. This study examines the attitudes of Palestinian university students toward honor killings, with a focus on understanding how the younger, more educated generation perceives this phenomenon. Given the role of socialization and moral development in shaping beliefs, this research explores whether gender, geography, and religious background influence attitudes toward honor-based violence.

**Methods:**

A quantitative, cross-sectional survey was conducted among students at An-Najah National University, the largest university in the West Bank. A structured questionnaire, developed by the researchers, was distributed online to assess students’ attitudes toward honor killings, particularly concerning women’s marital status and involvement in perceived moral transgressions. Statistical analyses, including chi-square tests and logistic regression, were employed to examine associations between demographic factors (gender, geographical location, and religious affiliation) and students' responses.

**Results:**

Findings revealed that while a significant portion of students justified the killing of individuals engaging in extramarital sexual relationships, they largely opposed violence against women who had non-sexual interactions with men. Gender differences were evident, with male students exhibiting stronger endorsement of honor-based violence compared to females. Psychological constructs such as moral disengagement and cognitive dissonance may play a role in justifying or rejecting honor killings, with religious and cultural influences further shaping these attitudes.

**Conclusions:**

The study highlights the persistence of honor-based justifications for violence among segments of the younger generation, emphasizing the need for psychological and educational interventions. Addressing cognitive biases, reshaping social norms, and implementing policies that challenge gender-based violence are critical for fostering attitudinal change. The findings contribute to the broader discourse on honor crimes and gender equality in Palestinian society, offering insights for future research and policy development.

## Background

Research consistently documents that honor killings, as a severe form of gender-based violence, remain a grave threat to women’s lives in Palestine and across the Middle East. In Palestine, prevalence rates are difficult to establish due to underreporting and social stigma, but qualitative research and field data indicate that such killings are recurrent, with dozens of cases annually [1, 2]. The phenomenon is more pronounced in rural and conservative regions, where patriarchal structures are stronger and adherence to customary norms is rigid [2, 3]. Socioeconomic factors, limited access to legal recourse, and the entrenchment of tribal traditions contribute to both the justification and concealment of these acts [4, 5]. Demographically, young women are most at risk, particularly those who are perceived as violating codes of sexual conduct, choosing their own partners, or attempting to assert autonomy in marriage decisions [3]. Cases such as the killing of Israa Gharib underscore how the convergence of social media exposure, familial suspicion, and lack of legal protection can culminate in fatal violence, with the perpetrators often shielded by communal silence and sometimes even celebrated for their actions [1, 2].

Honor killing in Palestine is most commonly committed by immediate male relatives—fathers, brothers, and uncles—who are driven by the imperative to restore family reputation in the wake of alleged transgressions by female kin [3, 6]. The logic of collective honor is deeply embedded in oral traditions, proverbs, and social discourse, which simultaneously silence women and legitimize male authority over their lives and bodies [3]. The patriarchal ideology underpinning these acts is not only reinforced by familial dynamics but is also heightened by broader socio-political pressures, including the militarized context of occupation and nationalist anxieties [2]. In some instances, other relatives or community members may be complicit or directly involved, particularly when family members are seen as failing to act decisively to eliminate shame [4]. Intersectional analysis reveals that women from marginalized socioeconomic backgrounds or those lacking social support are especially vulnerable [1]. Ultimately, the persistence of honor killing reflects the entanglement of gender, power, and collective identity, with such violence positioned as both a product and a mechanism of patriarchal control [3, 6, 7].

In the Palestinian cultural context, the tradition values oral and aural modes of communication, highlighting the significance of spoken discourse while deeming written and visual forms as potentially deceptive and malevolent [8]. Psychological perspectives suggest this reliance on auditory communication in Palestinian culture contributes to the oppression and victimization of women, particularly evident in the alarming occurrence of honor killings. Due to the deeply ingrained psychological notions of honor and the importance placed on public and private reputation, the mere accusation or spread of gossip can serve as justification for male individuals to commit acts of violence against women so as to suppress public discussions surrounding gossip and rumors (kalam al-nas in Arabic)—the primary means through which society imposes the honor code on the individual [8]. Awwad points out that "gossip is a tool used by community' members to spread the unpleasant reality or truth that a certain family's honor has been tarnished, and therefore a family's social and prestigious status is in danger" ([9] p. 45). The Palestinian community exerts psychological pressure on the individual through public gossip to kill the supposedly transgressive female characters.

The Palestinian family is a political entity with deeply ingrained patriarchal and conservative norms that dictate social and political structures. These norms are enforced through both discursive and tangible means, ensuring strict societal and political standards. Psychological research shows that breaking these norms can lead to severe consequences, often resulting in the death of the transgressive family member [3]. The family has a hierarchical structure, with age and gender playing significant psychological roles in determining roles and responsibilities. The father holds absolute authority, and each member understands their place within the family from a psychological perspective [10–12].

Honor in Palestinian culture is based on sexual purity [7], with individuals avoiding shame from deviating from societal norms. Fadia Faqir, a Jordanian novelist and critic, highlights the psychological importance of female virginity and chastity in Arab societies. These qualities serve as a boundary between respect and shame, with families appointing their honor to the virginity of their unmarried daughters and the chastity of their married ones. Honor killings in Jordanian society are a significant issue in Arab societies. "Female violators of the honor code face a different fate; punishment in some form is inescapable" ([13] p. 69).

Faqir's analysis of honor crimes in Jordan is relevant to understanding similar social issues in Palestine [13]. Legal frameworks in Palestine, including the Jordanian Penal Code No. 16 of 1960 in the West Bank and the Mandate Penal Code No. 74 of 1936 in Gaza, are biased against women, leaving them vulnerable [14, 15]. Daher-Nashif [1] highlights the psychological neglect of police investigations into killings of women in Palestine due to a prevailing masculine mentality. Moreover, honor killings are often punished less severely than other forms of murder [16]. In Palestinian society, practices such as silence and the covering up of honor crimes are prevalent [4], demonstrating psychological avoidance mechanisms.

The discourse of honor holds significant sway in the Arab world, shaping psychological dynamics within families and affecting relationships with the broader community. Honor crimes involve the killing of women by relatives, motivated by the psychological belief that the woman's actions have dishonored the family, often through engagement in extramarital sexual behavior. Scholars like Abu Rabia ([5] p. 34) and Faqir ([13] p. 73) highlight how women who commit adultery, become pregnant out of wedlock, or their families often threaten face homelessness in the name of honor. Palestinian culture, influenced by patriarchal and pre-Islamic norms, reflects these psychological dynamics.

Rubenberg [2] highlights the institutionalization of misogyny and patriarchy in Middle Eastern culture, predating Islam's emergence. Kulczycki and Windle [17] note the prevalence of honor killings in Muslim countries but debate whether specific Islamic traditions support them. While some argue that strict sexual mores in Islamic law indirectly condone honor killings, the Qur'an sets high evidentiary standards for accusations of adultery, prohibiting baseless accusations and prescribing severe punishment for those who make them. Islam prohibits honor crimes, emphasizing the sanctity of human life and condemning unjust acts against women [10–12]. The Qur'an illustrates how Islam liberated women from pre-Islamic oppression, where practices like burying women alive were common. Islam emphasizes the psychological value of human life, equating the unjust killing of one person to the destruction of all humanity [10–12].

In the realm of honor-related actions, the discourse of honor shapes familial psychological dynamics and community interactions. Perpetrators of honor crimes, often male relatives, navigate dual psychological roles as both actors in their relationships and members of society. While honor loss typically drives such crimes in the Arab world, the concept of "shame" is pivotal, especially in honor-centric cultures like Palestine [5]. According to Abu Rabia ([5] p. 34), a woman's misbehavior diminishes her male relative's 'ird (honour which is linked to female sexual purity), leading to a psychological quest for 'ird restoration. Deviations from societal norms are deemed shameful, with men feeling psychologically responsible for upholding honor, while women's actions are seen as tarnishing it. Instances of adultery or pregnancy out of wedlock may lead families to threaten women and girls for allegedly dishonoring them [13]. Honor crimes, involving the killing of women by relatives due to perceived shame brought upon the family, stem from the psychological belief that extramarital sexual behavior tarnishes the family's honor.

### Theoretical framework and review of literature

The Social Norms Theory in psychology sheds light on the root causes of honor crimes, emphasizing how societal norms shape individual behavior. Social norms encompass psychological beliefs about socially approved or disapproved actions within a specific group. Through this lens, honor killings are understood as influenced by cultural values, social conventions, and group identity [18–20]. Hasisi and Bernstein [4, 21] note that honor crimes often stem from perceived violations of socially acceptable norms, particularly concerning women's sexual behavior. Unlike more common forms of violence, like domestic and intimate partner violence, honor crimes are culturally specific [22]. These crimes originate from entrenched cultural expectations that prioritize family honor and community reputation, psychologically motivating and justifying their commission [23].

Honor crimes in Palestine reflect deeply rooted gender disparities and discriminatory practices within patriarchal societies. An intersectional psychological analysis reveals their disproportionate impact on women and girls, subjecting them to heightened vulnerability and harm in the name of preserving honor [24, 25]. When women challenge traditional gender roles and assert autonomy, it threatens male authority and societal norms [6, 26]. Perceived violations of cultural norms and family honor often trigger these crimes, psychologically reinforcing societal expectations and deterring behavior that challenges established standards [27, 28].

### Purpose of the study

This study aims to understand the attitudes of university students in Palestine towards honor killings and other manifestations of violence driven by notions of honor. It will examine the factors that contribute to the prevalent occurrence of honor killings and honor-based violence, as well as explore the level of acceptance towards the killing of women engaged in honor-shame. The researchers will propose strategies that help eradicate this phenomenon from society. The researchers will answer the following questions: The researchers will answer the following questions: (1) How do Palestinian university students generally feel about honor killing? (2) Are there statistically significant differences in attitudes toward honor killing and honor-based violence among study participants that can be attributed to various demographic variables (e.g., gender, year of study, faculty in which they study, place of residence, religion, marital status, and number of sisters)? (3) What are these students' perspectives on the phenomenon in terms of causes and strategies for eradicating it completely?

## Materials and methods

### Setting

Participating in this study were all college students attending a local Palestinian university in the West Bank during the 2022–2023 academic year. The university encompasses nine main faculties (Humanities, Economics and Social Sciences, Sciences, Educational Sciences and Teachers’ Training, Medicine and Health Sciences, Law, Islamic Shari’ah, Engineering and Information Technology, and Graduate Studies) distributed into two campuses. Despite having the same nationality—Palestinians—they differ in their religious beliefs, the number of siblings in their families, fields of study, places of residence, and marital statuses, which, as we will see, affect their attitudes towards honor killing.

### Participants

The study population consisted of college students enrolled at a Palestinian university located in the West Bank during the 2022–2023 academic year. A total of 178 students participated in the study, comprising 52 men (29.2%) and 126 women (70.8%). As detailed in Table 1, the distribution of participants across faculties was as follows: 23 students from the Faculty of Humanities, 11 from the Faculty of Economics and Social Sciences, 40 from the Faculty of Sciences, 4 from the Faculty of Educational Sciences and Teachers’ Training, 68 from the Faculty of Medicine and Health Sciences, 8 from the Faculty of Law, 2 from the Faculty of Islamic Shari’ah, 20 from the Faculty of Engineering and Information Technology, and 2 from the Faculty of Graduate Studies.

**Table 1:** Descriptive statistics for the demographic variables

Variable Level			DIR1		DIR2		DIR3		DIR4	
		N	M	SD	M	SD	M	SD	M	SD
Gender	Male	52	3.80	.78	2.69	1.05	2.42	.86	3.88	.80
	Female	126	4.08	.52	2.69	.95	2.32	.72	4.28	.60
Yearof study	Freshman (1st year)	15	4.04	.63	2.68	1.02	2.38	.74	4.19	.75
	Sophomore (2nd year)	50	4.14	.53	2.66	1.03	2.25	.73	4.24	.63
	Junior (3rd year)	56	3.98	.63	2.78	.98	2.39	.80	4.17	.68
	Senior (4th year)	42	3.98	.59	2.69	.92	2.38	.71	4.13	.64
	Super senior (5th or 6th year)	6	3.65	.84	2.21	.91	2.36	.96	4.03	1.01
	Graduate Student	9	3.62	.87	2.65	1.00	2.50	.96	3.83	.94
Faculty	Humanities	23	4.14	.62	2.38	1.11	2.18	.72	4.37	.71
	Economics	11	3.87	.57	2.35	1.04	2.23	.90	4.03	.46
	Sciences	40	3.95	.59	2.83	.74	2.40	.68	4.05	.66
	Educational Sciences	4	3.40	.48	2.59	1.08	2.71	.81	3.50	.38
	Medicine and Health Sciences	68	4.03	.65	2.74	.96	2.37	.71	4.22	.67
	Law	8	4.13	.56	2.73	.79	2.29	.81	3.96	.84
	Islamic Shari’ah	2	3.38	.54	2.88	.53	2.83	.71	3.58	.35
	Engineering and IT	20	4.07	.62	2.74	1.35	2.24	1.02	4.31	.71
	Graduate Studies	2	3.81	1.14	3.00	.00	3.42	.12	3.92	1.53
Place of residence	City	70	3.99	.64	2.48	.96	2.16	.70	4.24	.69
	Village (Small town)	76	4.06	.59	2.99	.95	2.46	.77	4.17	.66
	Refugee Camp	8	3.31	.67	2.47	.57	2.69	.62	3.56	.71
	Green Line Area	24	4.09	.53	2.43	.98	2.44	.87	4.09	.66
Religion	Islam	156	4.08	.58	2.73	.98	2.31	.72	4.25	.65
	Christianity	8	3.57	.89	2.09	1.06	2.23	1.12	3.67	.53
	Samaritans	7	3.14	.34	3.16	.20	3.19	.22	3.14	.33
	Secular (Atheist)	7	3.64	.38	2.16	.96	2.48	1.15	3.71	.73
Marital Status	Single	149	4.08	.58	2.65	.98	2.26	.71	4.24	.66
	Married	12	3.82	.79	2.92	1.13	2.88	1.06	3.94	.80
	Engaged	12	3.53	.68	3.00	.81	2.78	.65	3.64	.55
	Divorced (Separated)	5	3.38	.53	2.80	.68	2.83	.85	3.47	.61
Number of Brothers/sisters	One	31	4.02	.76	2.90	1.10	2.31	.79	4.20	.67
	Two	50	4.11	.53	2.53	.98	2.23	.72	4.33	.67
	Three	33	4.07	.54	2.73	.97	2.46	.82	4.15	.69
	Four	27	3.91	.59	2.42	.75	2.16	.63	4.03	.59
	Five	19	3.86	.53	2.75	.99	2.48	.65	4.13	.67
	Six or more	18	3.82	.85	3.06	.95	2.69	.94	3.86	.83

In the current study, DIR stands for Domain, referring to the four main thematic categories assessed through the survey. Each domain (DIR1 to DIR4) consists of multiple items measured on a 5-point Likert scale ranging from 1 (Strongly Disagree) to 5 (Strongly Agree). For each respondent, domain scores were calculated by averaging their responses to all items within the corresponding domain. DIR1 includes 13 items related to the perceived causes of honor crimes; DIR2 includes 8 items measuring support for killing perpetrators of honor-shame scandals; DIR3 consists of 6 items addressing attitudes toward killing female perpetrators; and DIR4 includes 7 items evaluating support for strategies to reduce honor crimes. The descriptive statistics for the entire sample (*N* = 178) are as follows: DIR1 ranged from 3.14 to 4.27, with a mean of 4.02 (SD = 0.63); DIR2 ranged from 2.09 to 3.17, with a mean of 2.68 (SD = 0.98); DIR3 ranged from 1.83 to 4.37, with a mean of 2.37 (SD = 0.75); and DIR4 ranged from 3.14 to 4.54, with a mean of 4.16 (SD = 0.68). These scores reflect the level of agreement with the items in each domain and were used for the demographic comparisons presented in Table 1 above

In terms of residential distribution, 39.3% of participants resided in urban areas (cities), 42.7% in rural villages, 0.05% in refugee camps, and 13.48% in other rural settings. Regarding religious affiliations, 87.64% identified as Muslim, 4.5% as Christian, 3.9% as Samaritan, and 3.9% reported no religious affiliation. Marital status varied as well, with 83.7% identifying as single, 6.74% as married, 6.74% as engaged, and 2.8% as divorced or separated.

Family size, specifically the number of sisters per participant, was also documented. Among the respondents, 17.4% had one sister, 28% had two sisters, 18.5% had three sisters, 15.1% had four sisters, 10.67% had five sisters, and 10.1% had six or more sisters. These demographic variables were considered critical for analyzing the participants' attitudes toward honor killings, as they reflect the intersectionality of social, cultural, and familial dynamics within Palestinian society.

The overall population for this study comprises all students enrolled at the university, which includes more than 20,000 students across various faculties and departments. The target population was not limited to a specific faculty but represented the broader student body. A random sample of 300 students was initially selected to ensure diversity and minimize sampling bias. However, due to the highly sensitive and stigmatized nature of the topic—honor killings—only 178 of the returned surveys were deemed valid and suitable for analysis. The reduced number of usable responses was influenced by several factors, including the cultural sensitivity of the subject, fear of social condemnation or legal repercussions, and general reluctance to discuss honor-related issues. These limitations, combined with logistical constraints and access challenges, contributed to the final sample size.

### Materials

To quantitatively examine students’ perceptions and attitudes toward honor killing and honor-based violence, the researchers designed a comprehensive online questionnaire. An initial pool of sixty declarative statements was developed, with approximately one-third addressing perceived causes of honor killing, another third focusing on strategies for mitigating the phenomenon, and the remainder exploring students’ levels of acceptance of such violence. The instrument underwent content validation by a panel of seven expert reviewers, all of whom held doctoral degrees and academic positions with research expertise.

The questionnaire was organized into four subsections, and the reviewers employed the weighted average method to facilitate logical and efficient analysis of the data. Items with a correlation coefficient below 0.6 were eliminated. Mean scores for the upper and lower quartiles were calculated to identify items demonstrating high discriminatory power. Furthermore, a t-test for two independent samples was applied, and items lacking statistically significant differences were excluded. This process yielded a refined instrument comprising 34 items addressing key themes: causes of honor killing, acceptance of killing individuals involved in honor-shame incidents irrespective of gender, acceptance of killing women in such situations specifically, and strategies to prevent honor killing.

The Kaiser–Meyer–Olkin (KMO) test confirmed the factorial validity of the questionnaire, with a value of 0.955 indicating strong suitability for exploratory factor analysis. The principal component method, combined with oblimin rotation, was used to remove orthogonal items, eliminating those with a communality or factor loading below 0.3. Ultimately, 34 items were retained. Responses to these items were measured on a five-point Likert scale, ranging from "strongly agree" (5) to "strongly disagree" (1). The reliability of the instrument was assessed using Cronbach’s alpha coefficient, which yielded a value of 0.86, indicating high internal consistency, as it exceeded the commonly accepted threshold of 0.7.

### Data collection and analysis

The questionnaire was administered online and translated into Arabic, the participants’ native language, to ensure clarity and accuracy of responses. A total of 178 completed surveys were collected for analysis. Students' general attitudes toward honor killing were assessed using the five-point Likert scale, with score intervals categorized as follows: 4.2–5 indicating a very high level of agreement, 3.4–4.19 denoting a high level, 2.6–3.39 reflecting a moderate level, 1.8–2.59 indicating a low level, and scores below 1.8 representing a very low level of agreement. Descriptive statistics, including means and standard deviations, were calculated to analyze the data.

To determine whether students’ attitudes significantly differed from established threshold scores (particularly 4.2 and 3.4), a one-sample t-test was conducted. The thresholds for interpreting Likert-scale scores were determined by dividing the scale into equal intervals, following standard psychometric guidelines [29]. Hypothesis testing was used to examine the significance of differences in mean scores. Additionally, stepwise multiple regression analysis was performed to explore the influence of demographic variables — including religion, marital status, and gender — on students’ attitudes toward honor killing and their perspectives on strategies to reduce its occurrence.

## Results

### Students’ attitudes toward honor killing

The analysis of students’ general attitudes toward the causes of honor killings indicated a high level of perceived significance regarding the factors contributing to such crimes within Palestinian society. As presented in Table 2, the responses yielded a mean score of 4.00 with a standard deviation of 0.62. The statistical analysis demonstrated that students’ perceptions of the causes of honor crimes did not reach the "very high" threshold (a score of 4.2). Specifically, the results of the one-sample t-test supported the rejection of the null hypothesis when the students’ responses were compared against the very high threshold, indicating a statistically significant difference. Conversely, when the responses were compared to the "high" threshold (3.4), the t-test results confirmed a significant alignment with this level, suggesting that students’ attitudes toward the causes of honor killing were consistent with a high, though not very high, level of agreement.

**Table 2:** One sample t-test for students’ attitudes toward honor killing

				Test Value = 4.2		Test Value = 3.4		Test Value = 2.6		Test Value = 1.8	
	M	S D	df	T	P	T	P	T	P	t	P
DIR1	4.00	0.62	177	-4.26	0.00	12.92	0.00				
DIR2	2.69	0.97	177	-20.65	0.00	-9.69	0.00	1.27	0.21		
DIR3	2.35	0.76	177	-32.36	0.00	-18.36	0.00	-4.36	0.00	9.65	0.00
DIR4	4.16	0.68	177	-0.78	0.44						
TOTAL	3.41	0.48	177	-22.07	0.00	0.36	0.72				

Regarding to the second dimension of the survey, which examined the acceptance of killing individuals involved in honor-shame scandals regardless of gender, the analysis revealed that the mean response (M = 2.6) closely reflect the medium threshold. The corresponding p-value (0.21) and t-test statistic (1.27) did not support the rejection of the null hypothesis, indicating that participants' attitudes did not significantly differ from the predefined medium level of acceptance.

In contrast, when examining participants’ attitudes toward the acceptance of killing only female perpetrators involved in honor-shame scandals, the mean response was lower (M = 2.35; SD = 0.76), reflecting generally negative attitudes toward this practice. The mean score fell between the "medium" and "low" thresholds, suggesting that respondents largely rejected the justification of killing female perpetrators in honor-related incidents.

Concerning strategies for reducing honor killings, participants demonstrated overwhelming support for interventions aimed at eradicating the phenomenon. The mean score for this dimension fell within the "very high" category (M = 4.2), indicating strong endorsement of efforts to eliminate honor killings within Palestinian society.

As shown in Table 3, participants identified several key causes of honor crimes. The absence of effective and deterrent legal measures against perpetrators was perceived as the most influential factor (M = 4.46), followed by a perceived decline in religious commitment within certain segments of the community (M = 4.27). Although poverty was acknowledged as a contributing factor, it was ranked as the least influential cause (M = 3.17) relative to other factors identified in the study.

**Table 3:** Means of survey items in each domain

No	Item	Mean
First Domain: Causes of honor crimes		
1	Absence of religious faith among a large segment of the society	4.27
2	Absence of deterrent legislations and laws against perpetrators of honor crimes	4.46
3	Legal loopholes of which the perpetrators of these crimes take advantage	4.08
4	Lack of interest by police and security services in handling complaints from women	4.08
5	Poor culture and education among people mainly males	4.28
6	Excessive adherence to family honor	4.03
7	Adherence to prevailing community customs and traditions	4.22
8	Poverty and deteriorating economic conditions	3.17
9	Family disintegration and the absence of follow-up and oversight of sons and daughters	4.06
10	Incorrect socialization in which men and women are segregated in a conservative society	4.17
11	Indirect support of these crimes by a significant portion of society's notables and extremist clerics	3.69
12	The desire to cover up the crimes of harassment and adultery	4.13
13	Absence of religious faith	3.39
Second Domain: Killing perpetrators of honor-shame scandals		
1	Married couples (men and women) who engage in lewd behavior should be put to death	2.70
2	Married couples (men and women) who date or associate with other men or women ought to be put to death	2.69
3	Any single person who engages in interpersonal sex should be executed, male or female	2.29
4	A single person who is dating or making friends with others should be put to death, male or female	2.61
5	If a single person (male or female) engages in an immoral sexual relationship with a member of their religion, that person must be put to death	2.66
6	If a single person (male or female) engages in an immoral sexual relationship with someone who is not a member of their religion, that person must be put to death	3.17
7	If a married person (male or female) engages in an immoral sexual relationship with a member of their religion, that person must be put to death	3.16
8	If a married person (male or female) engages in an immoral sexual relationship with someone who is not a member of their religion, that person must be put to death	2.25
Third Domain: Killing female perpetrators of honor-shame scandals		
1	If I find out that my sister is having an immoral sexual relationship with another man, I will kill her	1.94
2	If I find out that my sister is befriending other men without having sex with them), I will kill her	2.15
3	If I find out that one of my female relatives is having an immoral sexual relationship with another man, I will kill her	1.93
4	If I found out that one of my female relatives befriended other men without having sexual relations with them, I would kill her	1.89
5	If I find out that a girl/female who is not my relative is having a lewd sexual relationship with another man, I will kill her	1.83
6	If I find out that a girl/female who is not my relative is befriending other men without having sexual relations with them, I will kill her	4.37
Fourth Domain: Reducing honor crimes		
1	Applying the provisions of Islamic law with regard to crimes of adultery, defamation and harassment	3.88
2	Eliminating any trace of nepotism or drop the personal right in family murders	4.48
3	Ensuring that the perpetrators of these crimes do not go unpunished	3.96
4	Canceling the role of clans and reform committees in intervening to resolve these issues	3.88
5	Confidentiality in dealing with honor killings by the security and police authorities	4.22
6	Repealing laws and legal texts that are lenient toward honor crimes and may indirectly encourage violence against women.	4.54
7	Educating the public about the true meaning of honor	4.18

Regardinf to participants' attitudes toward punishment for individuals involved in honor-shame scandals, the findings indicated a general tendency to support severe penalties in cases of immoral sexual relationships conducted outside one’s religious group. Specifically, participants agreed with the execution of individuals engaged in such relationships, with a mean score of 3.17. Similar attitudes were observed toward married individuals involved in illicit relationships, regardless of whether these relationships occurred within or outside their religious group, although the preference for execution was notably lower in cases involving relationships outside one’s religion (M = 2.25).

As expected, participants expressed strong disagreement with the notion of killing unrelated females for merely befriending men without engaging in sexual relationships, as evidenced by a high mean score (M = 4.37). However, attitudes shifted when the female in question was a relative of the perpetrator, with participants demonstrating a higher level of acceptance for punishment in such cases (M = 1.83).

Participants also overwhelmingly supported legislative and social reforms aimed at combating violence against women and addressing honor crimes. There was particularly strong support for repealing legal texts that encourage violence against women (M = 4.54) and eliminating nepotism and favoritism in family-related murder cases (M = 4.48). Additional strategies perceived as effective included applying Islamic law provisions concerning adultery and defamation, as well as limiting the influence of clans in resolving such crimes, with mean scores of 3.88 for both strategies.

### Impact of demographic variables on attitudes

In addressing the second research question, which sought to explore the impact of demographic variables on participants’ attitudes toward honor killings, the results presented in Table 4 revealed significant differences based on religion, marital status, and gender.

**Table 4:** Stepwise multiple regression for the effect of variables on the study domains

	model		B	T	P	R	R^2^	F	P
Total	1	(Constant)	3.61	51.30	0.00	.235^a^	0.05	10.24	0.00
		RELIGION	-0.16	-3.20	0.00				
DIR1	1	(Constant)	4.33	47.93	0.00	0.30	0.09	17.09	0.00
		RELIGION	-0.26	-4.13	0.00				
	2	(Constant)	4.46	43.34	0.00				
		RELIGION	-0.19	-2.76	0.01	0.35	0.12	12.25	0.00
		MARITAL STATUS	-0.18	-2.62	0.01				
	3	(Constant)	4.06	19.53	0.00				
		RELIGION	-0.16	-2.33	0.02	0.38	0.15	9.97	0.00
		MARITAL STATUS	-0.18	-2.67	0.01				
		GENDER	0.22	2.21	0.03				
DIR2									
DIR3	1	(Constant)	2.01	17.56	0.00	0.25	0.06	11.38	0.00
		MARITAL STATUS	0.26	3.37	0.00				
DIR4	1	(Constant)	4.55	46.07	0.00	0.32	0.10	20.25	0.00
		RELIGION	-0.31	-4.50	0.00				
	2	(Constant)	3.97	17.84	0.00	0.38	0.14	14.71	0.00
		RELIGION	-0.27	-3.92	0.00				
		GENDER	0.31	2.89	0.00				
	3	(Constant)	4.11	18.25	0.00	0.42	0.18	12.45	0.00
		RELIGION	-0.19	-2.58	0.01				
		GENDER	0.31	2.97	0.00				
		MARITAL STATUS	-0.19	-2.63	0.01				

Religion emerged as a key determinant of attitudes, with Muslim participants providing more justifications for honor killings compared to respondents from other religious affiliations. Moreover, Christian participants cited more justifications than Samaritans, atheists, or those with no religious affiliation.

Marital status was also found to influence attitudes significantly. Unmarried respondents reported more reasons for honor killings than their married, engaged, or divorced counterparts. Additionally, gender differences were observed, with female participants reporting a higher number of justifications for honor crimes compared to male participants.

In relation to the second domain, which examined participants’ acceptance of killing individuals involved in honor-shame scandals regardless of gender, the analysis revealed no statistically significant effect for any of the examined demographic variables. This indicates a general consensus among participants, irrespective of their demographic characteristics, regarding their attitudes toward this issue.

In the third domain, which focused specifically on the acceptance of killing only female perpetrators involved in honor-shame scandals, marital status emerged as a significant factor influencing participants’ attitudes. Divorced respondents exhibited a higher level of acceptance toward the killing of female perpetrators compared to their engaged, married, or single counterparts. Engaged participants demonstrated greater acceptance than married respondents, while single participants reported the lowest level of acceptance within this domain.

With respect to the fourth domain, which addressed participants' views on strategies for reducing honor killings, the analysis demonstrated that religion, gender, and marital status significantly shaped participants’ responses. Muslim participants proposed a greater number of strategies to reduce honor killings compared to those from other religious affiliations. Additionally, female participants suggested more solutions than male participants. Marital status also played a role, with single respondents offering more strategies than married, engaged, or divorced participants, reflecting the diverse impact of demographic variables on attitudes toward solutions for addressing honor crimes.

It is important to note that certain demographic variables, including the year of study, faculty affiliation, place of residence, and the number of sisters in the family, did not exhibit any statistically significant effect on participants' attitudes across any of the four domains analyzed.

## Discussion

This article has explored the psychological perspectives of Palestinian students on honor killings, attributing them to various factors such as ineffective laws, inadequate penalties, and a lack of adherence to religious teachings. Palestinian legal systems, which are discriminatory, contribute to a diminished sense of moral accountability for offenders, potentially enabling them to commit honor crimes with impunity. The primary discriminatory legal provision facilitating impunity for honor crimes in Palestine is Article 340 of the Jordanian Penal Code No. 16 of 1960, which remains in effect in the West Bank. This article grants extenuating circumstances to men who commit violence against female relatives in so-called honor cases, often resulting in reduced sentences or acquittals. According to Hamamra [10], such legal frameworks, alongside the absence of robust protections in the criminal justice system, perpetuate a climate in which perpetrators are seldom held fully accountable. The United Nations Development Programme ([14] p. 27) also highlights that these discriminatory laws, together with social norms, undermine women’s security and impede efforts to address domestic violence in Palestine. These findings reflect previous studies [1, 15, 16], which argue that legal systems discriminate against women, leaving them vulnerable and devoid of protection from both the government and other criminals.

In the absence of robust legislation and effective legal sanctions serving as deterrents, coupled with a lack of adherence to Islamic teachings, the study found that a majority of participants believed individuals engaged in immoral sexual relationships should face death, regardless of gender. Social pressure and public scrutiny pressures Palestinians to execute supposedly transgressive female individuals [3]. Within Palestinian Middle Eastern culture, misogyny and patriarchy are deeply ingrained and justified philosophically and religiously [2]. However, Islam strictly forbids honor crimes, liberating women from the oppression of the pre-Islamic era where they were buried alive to spare men embarrassment (Qur'an, 16:58–59). Islam emphasizes the psychological sanctity of human life, stating that killing one innocent person is akin to killing all of humanity (Qur'an 5:32; 6:151; 17:33; 4:93). Consequently, the majority of participants opposed the killing of girls or women engaging in non-sexual friendships with men, though they supported the idea of killing female relatives involved in sexual relationships with men.

Importantly, this study recognizes that honor-based violence cannot be understood solely through the lens of cultural specificity. The Israeli occupation plays a significant role in shaping the structural conditions under which gender-based violence occurs in Palestine. The occupation has severely fragmented the Palestinian legal system, eroded the rule of law, and weakened institutional accountability—conditions that exacerbate impunity for perpetrators and hinder protection for women. The daily realities of military checkpoints, settler violence, displacement, economic deprivation, and political instability place additional stress on family and social structures, often amplifying patriarchal control as a form of coping or social regulation.

These overlapping systems of domination—including colonial occupation, patriarchy, class inequality, and legal fragmentation—form the backdrop against which honor crimes persist. Adopting a critical and intersectional perspective allows for a more holistic understanding of how gender, class, and politics intersect to reproduce and sustain violence against women in Palestine.

Based on the study's findings, the most effective psychosocial approaches for preventing honor crimes include abolishing the discriminatory laws that endorse violence against women and prohibiting nepotism [16]. While Palestinian laws do not formally endorse violence against women, certain legal provisions remain lenient toward perpetrators of honor-based violence, particularly when cultural or familial motives are cited. For example, Article 18 of the Jordanian Penal Code (still in effect in parts of the West Bank) and some remnants of British Mandate and Egyptian law in Gaza have historically allowed reduced sentences for crimes committed in a “fit of fury” or under the pretext of defending family honor. These legal loopholes do not criminalize VAW comprehensively, and in some cases, they implicitly condone or mitigate the consequences of gender-based violence, contributing to a climate of impunity.

By implementing these strategies, the legal framework can better reflect principles of non-discrimination, human rights, and gender equality. However, it's crucial to acknowledge that legal changes alone may not suffice to fully address the issue of honor crimes. A comprehensive psychological approach is necessary, involving targeted interventions to challenge harmful cultural norms and attitudes, such as settling matters internally via tribalism exerted by clan leaders and reform committees [4]. Educational programs, public awareness campaigns, and active community engagement are also vital in fostering lasting change and promoting a society that prioritizes the rights and well-being of all individuals.

Reducing nepotism stands out as a highly effective strategy in preventing honor crimes. Nepotism perpetuates psychological power imbalances within families and communities, allowing certain individuals to dominate and control others [10]. This imbalance increases the risk of violence against women, including honor crimes. Importantly, women are often disproportionately affected by nepotism, limiting their opportunities for leadership and decision-making in social and familial contexts. Addressing nepotism can break down barriers to gender equality, promoting a more inclusive society where women can participate actively and assert their psychological agency.

Demographic factors like gender, religion, and marital status influence the cognitive attitudes of Palestinian students towards honor crimes. Islam, a religion that emphasizes human life's sanctity and equality, has the potential to reduce honor-based violence in Palestine [10, 11]. To challenge patriarchal norms and promote women's rights and empowerment, it is crucial to promote a deeper understanding of Islamic teachings and actively advocate for women's rights within the Palestinian context. Additionally, religion plays a complex and influential role in shaping attitudes toward honor crimes. While religious teachings, particularly within Islam, explicitly condemn unjust violence and emphasize the sanctity of life, cultural interpretations and patriarchal norms often distort these teachings to justify violence against women [12].

The attitudes towards women involved in honor-shame scandals can be influenced by their marital status, with single or unmarried women being more susceptible due to societal expectations surrounding virginity and traditional values. In honour-shame cultures like Palestine, “single” typically means never-married, while “unmarried” refers to women who are single, divorced, or widowed—though never-married women often face the greatest social scrutiny regarding honour and virginity [10–12, 30]. Punishment may be seen as a behavioral mechanism for maintaining marital harmony and restoring lost family honor. Women who engage in dishonorable behaviors, such as divorce or widowhood, may face heightened scrutiny and betrayal of marital status expectations, leading to harsher responses and potentially punitive measures [10, 11]. This is particularly true in societies where the preservation of family honor and the woman's role as a wife and mother are paramount. This reflects [6, 26] who postulate that challenging or deviating from traditional gender roles, women have the potential to be perceived as a threat to established societal norms and male authority and, consequently, be victims of honor-based violence.

## Conclusion

This study examines the attitudes of Palestinian university students toward honor killings, highlighting the complex influence of cultural, religious, and gender norms. The findings show that many participants supported killing individuals engaged in sexual relationships considered immoral within their community, regardless of gender. However, most opposed killing women solely for forming non-sexual friendships with men, while maintaining support for killing female relatives involved in sexual relationships. This reflects the persistence of patriarchal notions of family honor in Palestine, where women often bear a disproportionate responsibility for upholding family reputation.

The study suggests that addressing honor crimes in Palestine requires repealing legal provisions that offer leniency to perpetrators and dismantling nepotism and tribal loyalties that hinder justice. These recommendations are particularly relevant given the historical legacy of laws and social structures that continue to enable honor-based violence in Palestinian society.

Several targeted interventions can help reduce honor killings among Palestinian youth. These include integrating gender equality and human rights education into university curricula, launching peer-led dialogue programs that challenge harmful norms, and implementing awareness campaigns on campus and social media to denounce honor-based violence. Collaborating with religious leaders to counter misinterpretations that justify such acts is also essential. Additionally, empathy and bystander training, along with clear university policies and support services for at-risk students, can foster a safer, more informed, and non-violent campus environment.

The findings also indicate that Muslim participants identified more reasons for honor crimes, pointing to the role of religious interpretations in shaping attitudes. This underscores the need for targeted interventions within specific communities and the involvement of religious leaders in promoting rights-based interpretations of Islamic teachings.

Moreover, the study found that divorced respondents were more accepting of killing female relatives involved in honor-shame scandals. This highlights how social stigma and marginalization intersect with attitudes toward violence, especially in a context where divorced individuals often face heightened social scrutiny.

### Study limitations and future research

Despite its valuable insights, this study has several limitations that should be acknowledged. Firstly, the sample was drawn exclusively from An-Najah National University—the largest in the West Bank—which, while significant, limits the generalizability of the findings. Students from different universities, particularly those in Gaza, East Jerusalem, and rural or marginalized areas, may hold varying attitudes shaped by their distinct socio-political, religious, and cultural environments. As such, future research should expand to include multiple institutions across diverse regions of Palestine to ensure a more representative and nuanced understanding of public opinion on honor killings.

Secondly, the highly sensitive and stigmatized nature of the topic may have led to social desirability bias, where participants underreported beliefs they perceived as socially unacceptable. This could have resulted in a partial or skewed portrayal of prevailing attitudes. Future studies might benefit from employing mixed-method approaches, such as combining anonymous surveys with in-depth qualitative interviews, to elicit more candid responses and uncover the deeper cognitive and emotional processes influencing attitudes toward honor-based violence.

Furthermore, the current study relied primarily on self-reported attitudes without examining actual behaviors, family dynamics, or community-level influences. Subsequent research could incorporate longitudinal designs or ethnographic fieldwork to explore how such attitudes are reinforced or challenged over time and in daily life. It would also be beneficial to examine the roles of media exposure, educational interventions, and religious discourse in shaping young people’s views.

Finally, while this study focused on university students, future research should also explore attitudes among other key demographic groups—such as parents, educators, religious leaders, and policy-makers—to identify points of intervention across multiple societal layers. This broader approach would help inform the design of targeted awareness campaigns, legal reforms, and culturally sensitive educational programs aimed at preventing honor killings and promoting gender justice in Palestinian society.

## Data Availability

Data is available from the corresponding author upon request.
